# Impact of the histologic grade of acute gastrointestinal graft-versus-host disease on outcomes in pediatric patients treated with allogeneic hematopoietic stem cell transplantation

**DOI:** 10.3389/fmed.2023.1231066

**Published:** 2023-08-08

**Authors:** Eun Sil Kim, Yiyoung Kwon, Yon Ho Choe, Mi Jin Kim, Keon Hee Yoo

**Affiliations:** ^1^Department of Pediatrics, Kangbuk Samsung Hospital, Sungkyunkwan University School of Medicine, Seoul, Republic of Korea; ^2^Department of Pediatrics, Samsung Medical Center, Sungkyunkwan University School of Medicine, Seoul, Republic of Korea; ^3^Department of Pediatrics, Inha University Hospital, Incheon, Republic of Korea; ^4^Department of Health Sciences and Technology, SAIHST, Sungkyunkwan University, Seoul, Republic of Korea; ^5^Cell & Gene Therapy Institute, Samsung Medical Center, Seoul, Republic of Korea

**Keywords:** graft-versus-host disease, gastrointestinal, histologic, hematopoietic stem cell transplantation, pediatric

## Abstract

**Introduction:**

Acute gastrointestinal graft-versus-host disease (GVHD) is a common life-threatening complication after hematopoietic stem cell transplantation (HCT). We aimed to investigate outcomes according to the clinical, endoscopic, and histologic severity of gastrointestinal GVHD in pediatric patients treated with allogeneic HCT.

**Methods:**

This retrospective cohort study included pediatric patients who underwent sufficient endoscopic and histopathologic evaluation for clinically suspected acute gastrointestinal GVHD between 2010 and 2020.

**Results:**

Fifty-one patients were included (male proportion, 68.6% [35/51]; median age at HCT, 6.4 years). When the patients were classified according to the histologic severity of gastrointestinal GVHD, the severe group had an earlier onset of GVHD symptoms and a higher proportion of patients with severe clinical gastrointestinal GVHD than the mild-to-moderate and “absent” groups. In Cox proportional hazards regression analysis, the groups with more severe clinical and histologic gastrointestinal GVHD showed a higher risk of non-relapse mortality (NRM). The 5-year overall survival (OS) rates were 58.3 and 36.4% in the mild-to-moderate and histologic gastrointestinal GVHD groups, respectively (*p* = 0.0384). Patients with higher clinical and histologic grades of gastrointestinal GVHD showed higher cumulative incidence of NRM.

**Discussion:**

Our results demonstrated that histologic severity of gastrointestinal GVHD is a relevant factor affecting OS and NRM, and patients with mild-to-moderate or severe histologic gastrointestinal GVHD have worse outcomes than patients without histologic GVHD. These findings support the importance of assessing the histologic grade in the diagnostic evaluation of patients with clinical gastrointestinal GVHD.

## Introduction

Allogeneic hematopoietic stem cell transplantation (HCT) is a potentially curative treatment for malignant hematologic diseases and non-malignant diseases such as inborn errors of immunity (1). The graft-versus-leukemia (GVL) effect mediated by alloreactive donor T cells allows the treatment of hematologic malignancies with HCT. However, although natural killer cells only exert GVL effects, alloreactive donor T cells can also cause graft-versus-host disease (GVHD) (2). Despite advances in human leukocyte antigen typing and the use of immunosuppressants after transplantation, acute GVHD remains a leading cause of mortality and morbidity after allogeneic HCT (3).

After the skin, the gastrointestinal tract is the second most commonly affected organ, accounting for approximately 50% of all GVHD cases (4, 5). Approximately 25% of pediatric patients develop acute gastrointestinal GVHD after HCT (6). Gastrointestinal GVHD is commonly classified into upper and lower gastrointestinal GVHD based on clinical symptoms, and these two forms of GVHD have different prognoses. Upper gastrointestinal GVHD is associated with a better response to treatment with systemic steroids, and has a lower impact on overall survival (OS) and non-relapse mortality (NRM) than does lower gastrointestinal GVHD (7). The lower gastrointestinal tract is the most important organ in determining treatment response and outcome (8, 9). Among patients with lower gastrointestinal GVHD, those with clinical grades 3–4 have a higher risk of mortality than those with clinical grades 1–2 (4). Although the risk of severe gastrointestinal GVHD is known to be relatively lower in children than in adults, severe gastrointestinal GVHD has a 2-year mortality rate of 45% in pediatric patients treated with HCT (4).

Gastrointestinal GVHD is difficult to diagnose based on clinical manifestations alone due to non-specific symptoms and confounding factors such as chemoradiation toxicity, adverse drug reactions, and enteric infections (10, 11). Therefore, endoscopic and histologic findings play an important role in the diagnosis of gastrointestinal GVHD (12). However, previous studies have not clearly elucidated which of the clinical, endoscopic, and histologic grades correlates most strongly with the prognosis of GVHD. In addition, because endoscopy is an invasive procedure, it is not routinely performed in children, resulting in few studies reporting on endoscopic and histologic findings in pediatric patients. Therefore, in this study, we aimed to evaluate the outcomes according to the clinical, endoscopic, and histologic grades of gastrointestinal GVHD in pediatric patients treated with allogeneic HCT.

## Methods

### Study population

This retrospective study enrolled all pediatric patients who underwent allogeneic HCT and were suspected of developing acute lower gastrointestinal GVHD at the Department of Pediatrics, Samsung Medical Center, between January 2010 and December 2020. We re-evaluated gastrointestinal tract biopsy specimens obtained from patients with clinical gastrointestinal GVHD and analyzed clinical data obtained from medical records.

Inclusion criteria were as follows: (i) age < 18 years at the time of allogeneic HCT; (ii) clinically suspected lower gastrointestinal GVHD within 180 days of allogeneic HCT; (iii) ileocolonoscopy ± esophagogastroduodenoscopy or sigmoidoscopy + esophagogastroduodenoscopy for acute gastrointestinal GVHD (13, 14); and (iv) available histopathologic results of lower endoscopic (ileocolonoscopy or sigmoidoscopy) biopsy specimens obtained from at least three different sites. Patients with infectious colitis diagnosed by stool examination (i.e., culture or polymerase chain reaction assay), those with cytomegalovirus colitis diagnosed histologically after endoscopic biopsy, or those with chemoradiation toxicity were excluded from the study.

Baseline demographic information and clinical data at the first endoscopy, including age, sex, primary disease, conditioning regimen, donor type, stem cell source, GVHD prophylaxis regimen, lower gastrointestinal GVHD symptoms, other organ involvement with GVHD, and laboratory results, were obtained from the patients’ electronic medical records. During the endoscopic evaluations, biopsy specimens for histopathologic analysis were obtained from abnormal lesions in all segments of the lower gastrointestinal tract.

This study was a retrospective chart review, and waiving consent would not adversely affect the rights of welfare of the patients. In addition, it was not practical to reconvene all patients to obtain consent for this study due to contact changes or death of some patients. This study was approved by the Institutional Review Board of Samsung Medical Center (file no. 2021–12-129) and was conducted in accordance with the Declaration of Helsinki.

### Diagnostic criteria and grading systems for gastrointestinal GVHD

Acute gastrointestinal GVHD was defined as the presence of symptoms, including the overlap syndrome, within 180 days of HCT. All patients were classified as having clinical gastrointestinal GVHD grade 1 or higher, as this study was conducted only in patients with a clinical diagnosis of gastrointestinal GVHD. Among these patients, those without endoscopic or histologic findings of gastrointestinal GVHD were classified as the “absent” group. In addition, patients with grade 1 and 2 gastrointestinal GVHD were classified into the mild-to-moderate group, and those with grade 3 and 4 gastrointestinal GVHD were classified into the severe group.

We considered only the clinical, endoscopic, and histologic grades determined at the time of initial diagnosis of acute gastrointestinal GVHD and at the time of initial endoscopic evaluation and biopsy. The diagnosis and grading of clinical gastrointestinal GVHD were based on the Mount Sinai Acute GVHD International Consortium (MAGIC) criteria for acute gastrointestinal GVHD (5). The clinical gastrointestinal GVHD grades were defined as follows: (i) Grade 1, stool volume of 10–19.9 ml/kg/day or stool frequency of 4–6 episodes/day; (ii) Grade 2, 20–30 ml/kg/day or 7–10 episodes/day; (iii) Grade 3, >30 ml/kg/day or >10 episodes/day; and (iv) Grade 4, severe abdominal pain with or without ileus or grossly bloody stool regardless of the stool volume.

The endoscopic grades of gastrointestinal GVHD were determined according to a previously published validated grading scale and were defined as follows (15): (i) Grade 1, loss of vascularity and/or mild erythema; (ii) Grade 2, moderate mucosal edema and/or erythema; (iii) Grade 3, severe edema, erosions, erythema, and/or bleeding; and (iv) Grade 4, bleeding, ulceration, and exudation (16).

The histopathologic threshold for the diagnosis of lower gastrointestinal GVHD was the detection of at least one apoptotic body in a crypt per biopsy specimen (17). The histologic grades of gastrointestinal GVHD were determined according to the criteria of Lerner et al. and were defined as follows (18): (i) Grade 1, increased apoptotic epithelial cells without crypt loss; (ii) Grade 2, isolated crypt loss; (iii) Grade 3, contiguous crypt loss; and (iv) Grade 4, diffuse crypt loss with mucosal denudation.

### Statistical analysis

Student’s *t*-test and the Wilcoxon rank-sum test were used for continuous variables, and the chi-squared test or Fisher’s exact test was used for categorical variables to statistically compare groups according to the histologic severity of gastrointestinal GVHD. Univariate and multivariate Cox proportional hazards regression analyses were performed to examine the association between mortality and other variables. Variables with *p <* 0.1 in the univariate analysis were included in the multivariate analysis using a stepwise selection procedure. Results are expressed as hazard ratios (HRs) with 95% confidence intervals (CIs). Kaplan–Meier analyses were used to calculate the rates of OS and the cumulative incidence of NRM, and the log-rank test was used to detect overall statistical differences in the estimates. In addition, Spearman’s correlation analysis was used to assess the association between the clinician-determined clinical grade and the pathologist-reported histologic grade. All statistical analyses were performed using Rex Software (version 3.0.3; RexSoft Inc., Seoul, Korea).

## Results

### Characteristics of patients and HCT

**Figure 1:**
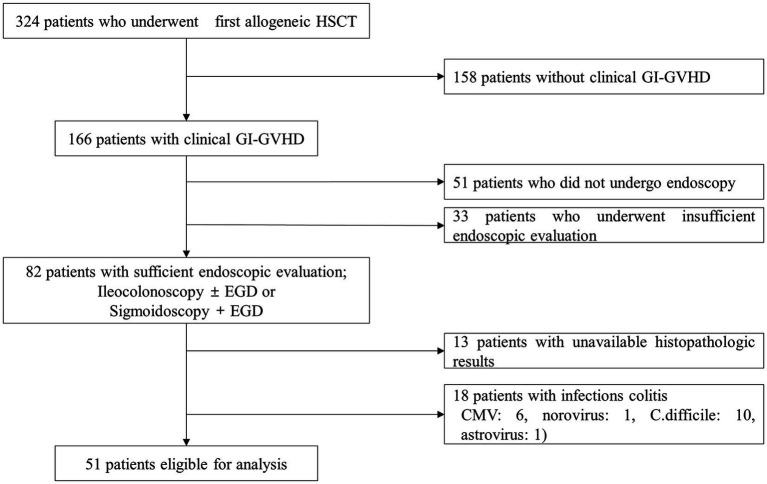
Flow diagram of the patient selection process. HCT, hematopoietic stem cell transplantation; GVHD, graft-versus-host disease; *C. difficile, Clostridioides difficile.*

During the study period, 324 consecutive pediatric patients underwent their first allogeneic HCT. Among these patients, 166 (51.2%) were diagnosed with clinical gastrointestinal GVHD and 82 underwent adequate endoscopic evaluation. After further exclusions according to our selection criteria, 51 patients were finally considered eligible for analysis (Figure 1).

The HCT characteristics of the 51 patients are shown in Table 1. The median age at HCT was 6.4 years, and 68.6% (35/51) of the patients were male. The most common primary disease category was hematologic malignancy (29/51, 56.9%), including acute lymphoblastic leukemia (18/51, 35.3%) and acute myeloid leukemia (6/51, 11.8%). The other primary disease categories were inborn errors of immunity (13/51, 25.5%), benign hematologic diseases (8/51, 15.7%), and miscellaneous diseases (1/51, 1.9%). Most patients (43/51, 84.3%) underwent myeloablative HCT, six patients (6/51, 11.8%) received a reduced-intensity conditioning regimen, and two patients (2/51, 3.9%) received no conditioning regimen. The stem cell sources were peripheral blood progenitor cells in 35 patients (68.6%) and cord blood in 16 patients (31.4%). Cyclosporine with methotrexate (23/51, 46.9%) was the most commonly used GVHD prophylaxis, followed by cyclosporine with mycophenolate mofetil (12/51, 24.5%), cyclosporine alone (6/51, 12.2%), tacrolimus with methotrexate (4/51, 8.2%), and tacrolimus with mycophenolate mofetil (4/51, 8.2%).

**Table 1:** Baseline characteristics of 51 pediatric patients with clinically suspected gastrointestinal GVHD.

Characteristics	Total (*n* = 51)
Age at HCT (years)	6.4 (3.1–12.6)
Sex, *n* (%)	
Male	35 (68.6)
Female	16 (31.4)
Primary disease, *n* (%)	
Hematologic malignancy	29 (56.9)
Benign hematologic disease	8 (15.7)
Inborn errors of immunity	13 (25.5)
Miscellaneous	1 (1.9)
Conditioning regimen, *n* (%)	
Myeloablative	43 (84.3)
Reduced intensity	6 (11.8)
None	2 (3.9)
Total body irradiation, *n* (%)	27 (52.9)
Donor, *n* (%)	
Matched unrelated	37 (72.6)
Haploidentical	7 (13.7)
Sibling	7 (13.7)
Stem cell source, *n* (%)	
Peripheral blood progenitor cells	35 (68.6)
Cord blood	16 (31.4)
GVHD prophylaxis, *n* (%)	
Tacrolimus + methotrexate	4 (8.2)
Tacrolimus + mycophenolate mofetil	4 (8.2)
Cyclosporine + methotrexate	23 (46.9)
Cyclosporine + mycophenolate mofetil	12 (24.5)
Cyclosporine alone	6 (12.2)
GVHD-involved organ except for the GI tract, *n* (%)	
Skin	46 (90.2)
Liver	21 (41.2)
GI alone	2 (3.9)
Maximum overall grade of acute GVHD, *n* (%)	
Grade 1	1 (2.0)
Grade 2	16 (31.4)
Grade 3	24 (47.1)
Grade 4	10 (19.6)

HCT, hematopoietic stem cell transplantation; GVHD, graft-versus-host disease; GI, gastrointestinal.

Most patients (49/51, 96.1%) had GVHD involving organs other than the gastrointestinal tract, most commonly the skin (90.2%). In addition, the maximum overall grade distribution of GVHD was grade 1 in 2.0%, grade 2 in 31.4%, grade 3 in 47.1%, and grade 4 in 19.6% of the patients.

### Correlation between the clinical and histologic grades of gastrointestinal GVHD

**Figure 2:**
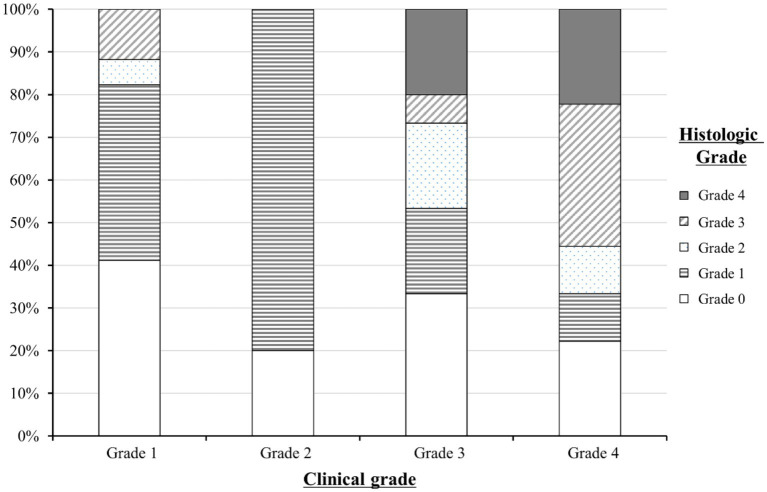
Distribution of the histologic grades of lower gastrointestinal graft-versus-host disease (GVHD) according to clinical grades. A total of 51 patients with clinical gastrointestinal GVHD were grouped into clinical grade 1 (*n* = 17), grade 2 (*n* = 10), grade 3 (*n* = 14), and grade 4 (*n* = 9). The distribution of histologic grades within each clinical grade is represented as a percentage on the *y*-axis.

The distribution of histologic grades according to clinical grades is summarized in Figure 2. Of the 51 patients, 22 (43.1%) had both upper and lower gastrointestinal symptoms and 29 (56.9%) had only lower gastrointestinal symptoms. At the time of the initial endoscopic evaluation, 17 patients (33.3%) had grade 1, 10 patients (19.6%) had grade 2, 15 patients (29.4%) had grade 3, and 9 patients (17.7%) had grade 4 clinical gastrointestinal GVHD.

The histologic grade was lower than the clinical grade in 35 of the 51 patients (68.6%). Grade 1 was the most common histologic grade in our patients (19/51, 37.3%), followed by grade 0 (negative biopsies; 16/51, 31.4%), grade 3 (6/51, 11.8%), grade 2 (5/51, 9.8%), and grade 4 (5/51, 9.8%). The Spearman’s correlation coefficient (ρ) between the clinical and histologic grades of lower gastrointestinal GVHD was 0.7 (*p* = 0.0052).

### Comparison of clinical characteristics according to the histologic severity of gastrointestinal GVHD

Of the 51 patients, 27 (52.9%) and 24 (47.1%) of them developed mild-to-moderate and severe clinical gastrointestinal GVHD, respectively. Patients developed acute gastrointestinal GVHD symptoms at a median of 24.0 days after HCT (interquartile range [IQR], 15.0–77.5 days). The median time from HCT to endoscopy was 52.0 days (IQR, 27.0–98.5 days), and the interval between the onset of gastrointestinal GVHD symptoms and endoscopy was 7.0 days (IQR, 4.0–17.0 days). The most common symptom observed at the time of endoscopy was diarrhea (100.0%), followed by abdominal pain (92.2%), hematochezia (21.6%), and nausea or vomiting (11.8%).

We categorized the patients into according to the severity of histologic gastrointestinal GVHD groups and compared their clinical characteristics. Compared with the absent and mild-to-moderate groups, the severe group showed statistically significant differences in the number of days from HCT to symptom onset (89.5 vs. 38.0 vs. 15.0 days, *p* = 0.028), the incidence of hematochezia (18.8% vs. 12.5% vs. 45.5%, *p* = 0.023), and the endoscopic severity of GVHD (*p* < 0.001). Other detailed results are shown in Table 2.

**Table 2:** Comparison of clinical characteristics according to the histologic severity of gastrointestinal GVHD.

Characteristics	Total	Absent (histologic grade 0)	Mild-to-moderate (histologic grades 1–2)	Severe (histologic grades 3–4)	*P*
No. of patients, *n* (%)	51 (100.0)	16 (31.4)	24 (47.1)	11 (21.6)	
Days from HCT to onset of symptoms, days (IQR)	24.0 (15.0–77.5)	89.5 (14.5–104.3)	38.0 (20.8–62.3)	15.0 (12.5–21.5)	0.028
Days from HCT to endoscopy, days (IQR)	52.0 (27.0–98.5)	97.0 (29.3–148.0)	53.0 (27.0–75.0)	27.0 (22.0–60.0)	0.082
Days from onset of symptoms to endoscopy, days (IQR)	7.0 (4.0–17.0)	9.5 (3.0–16.0)	6.5 (4.0–15.3)	7.0 (4.0–22.5)	0.986
Conditioning regimen, *n* (%)					0.180
Myeloablative	43 (84.3)	11 (25.6)	20 (46.5)	12 (27.9)	
Reduced intensity	6 (11.8)	3 (50.0)	3 (50.0)	0 (0.0)	
None	2 (3.9)	1 (50.0)	1 (50.0)	0 (0.0)	
Total body irradiation, *n* (%)	27 (52.9)	9 (33.3)	12 (44.4)	6 (22.2)	0.903
Donor type, *n* (%)					0.411
Matched unrelated	37 (72.6)	12 (75.0)	15 (62.5)	10 (90.9)	
Haploidentical	7 (13.7)	3 (18.8)	4 (16.7)	0 (0.0)	
Sibling	7 (13.7)	1 (6.3)	5 (20.8)	1 (9.1)	
Symptoms at endoscopy, *n* (%)					
Abdominal pain	47 (92.2)	14 (87.5)	22 (91.7)	11 (100.0)	> 0.990
Nausea or vomiting	6 (11.8)	3 (18.8)	2 (8.3)	1 (9.1)	0.088
Diarrhea	51 (100.0)	16 (100.0)	24 (100.0)	11 (100.0)	0.674
Hematochezia	11 (21.6)	3 (18.8)	3 (12.5)	5 (45.5)	0.023
Laboratory results at endoscopy					
Albumin (g/dl)	4.1 ± 0.4	4.0 ± 0.6	4.1 ± 0.4	4.3 ± 0.3	0.152
Total bilirubin (mg/dl)	0.3 (0.2–0.5)	0.3 (0.2–0.3)	0.4 (0.3–0.6)	0.3 (0.2–0.4)	0.068
Aspartate aminotransferase (U/L)	26.0 (20.0–31.0)	29.0 (24.0–33.8)	25.5 (20.0–35.0)	24.0 (19.0–28.0)	0.507
Alanine aminotransferase (U/L)	20.0 (13.0–42.0)	31.5 (14.3–46.0)	17.5 (13.8–43.8)	17.0 (11.5–27.0)	0.375
Clinical gastrointestinal GVHD grade, *n* (%)					0.127
Grades 1–2	27 (52.9)	9 (56.3)	16 (66.7)	2 (18.2)	
Grades 3–4	24 (47.1)	7 (43.8)	8 (33.3)	9 (81.8)	
Endoscopic gastrointestinal GVHD grade, *n* (%)					< 0.001
Absent (endoscopic grade 0)	2 (3.9)	2 (12.5)	0 (0.0)	0 (0.0)	
Grades 1–2	34 (66.7)	10 (62.5)	23 (95.8)	1 (9.1)	
Grades 3–4	15 (29.4)	4 (25.0)	1 (4.2)	10 (90.0)	
Antibiotics for ≥3 consecutive days*, n* (%)	29 (56.9)	9 (56.3)	13 (54.2)	7 (63.6)	0.934
Proton pump inhibitor*, n* (%)	8 (15.7)	2 (12.5)	2 (8.3)	4 (36.4)	0.1371

GVHD, graft-versus-host disease; HCT, hematopoietic stem cell transplantation; IQR, interquartile range.

### Factors associated with NRM

Relapse was confirmed in 7 of the 51 patients (13.7%). Univariate Cox proportional hazards regression analysis showed that the clinical, endoscopic, and histologic gastrointestinal GVHD grades were associated with NRM (Table 3). In multivariate Cox proportional hazards regression analysis, the groups with more severe clinical and histologic gastrointestinal GVHD showed a higher risk of NRM: higher clinical grade (severe vs. mild-to-moderate group: HR 13.02, 95% CI 2.70–62.78, *p* = 0.0014) and higher histologic grade (mild-to-moderate vs. absent group: HR 1.67, 95% CI 1.07–3.21, *p* = 0.0315; severe vs. absent group: HR 10.16, 95% CI 1.06–27.32, *p* = 0.0442; severe vs. mild-to-moderate: HR 2.92, 95% CI 1.61–3.43, *p* = 0.0287). However, the endoscopic severity of gastrointestinal GVHD was not associated with NRM (*p* > 0.05).

**Table 3:** Cox proportional hazards regression analysis of factors associated with non-relapse mortality.

	Univariable Cox analysis			Multivariable analysis with stepwise selection		
	HR	95% CI	*P*	HR	95% CI	*P*
Sex [female vs. male]	1.80	0.51–6.41	0.3056			
Age at HCT	1.04	0.96–1.14	0.3266			
Donor type						
[Matched sibling vs. haploidentical]	1.49	0.29–7.72	0.6368			
[Matched sibling vs. unrelated]	2.44	0.42–14.10	0.3176			
Albumin at HCT	0.73	0.23–2.35	0.5995			
Total bilirubin at HCT	2.35	1.34–4.14	0.4233			
AST at HCT	0.96	0.99–1.07	0.1055			
ALT at HCT	0.97	1.00–1.03	0.1081			
Clinical gastrointestinal GVHD grade						
[Grades 3–4 vs. 1–2]	2.90	1.10–7.64	0.0315	13.02	2.70–62.78	0.0014
Endoscopic gastrointestinal GVHD grade						
[Grades 1–2 vs. 0]	0.49	0.06–3.91	0.5038			
[Grades 3–4 vs. 0]	1.53	0.19–12.23	0.6908			
[Grades 3–4 vs. 1–2]	3.95	1.40–11.20	0.0096	3.57	0.12–12.62	0.5243
Histologic gastrointestinal GVHD grade						
[Grades 1–2 vs. 0]	2.03	1.07–5.28	0.0227	1.67	1.07–3.21	0.0315
[Grades 3–4 vs. 0]	4.66	1.32–16.49	0.0169	10.16	1.06–27.32	0.0442
[Grades 3–4 vs. 1–2]	2.45	1.13–5.29	0.0226	2.92	1.61–3.43	0.0287

HCT, hematopoietic cell transplantation; GVHD, graft-versus-host disease; AST, aspartate aminotransferase; ALT, alanine aminotransferase; HR, hazard ratio; CI, confidence interval.

### OS and cumulative incidence of NRM according to gastrointestinal GVHD

**Figure 3:**
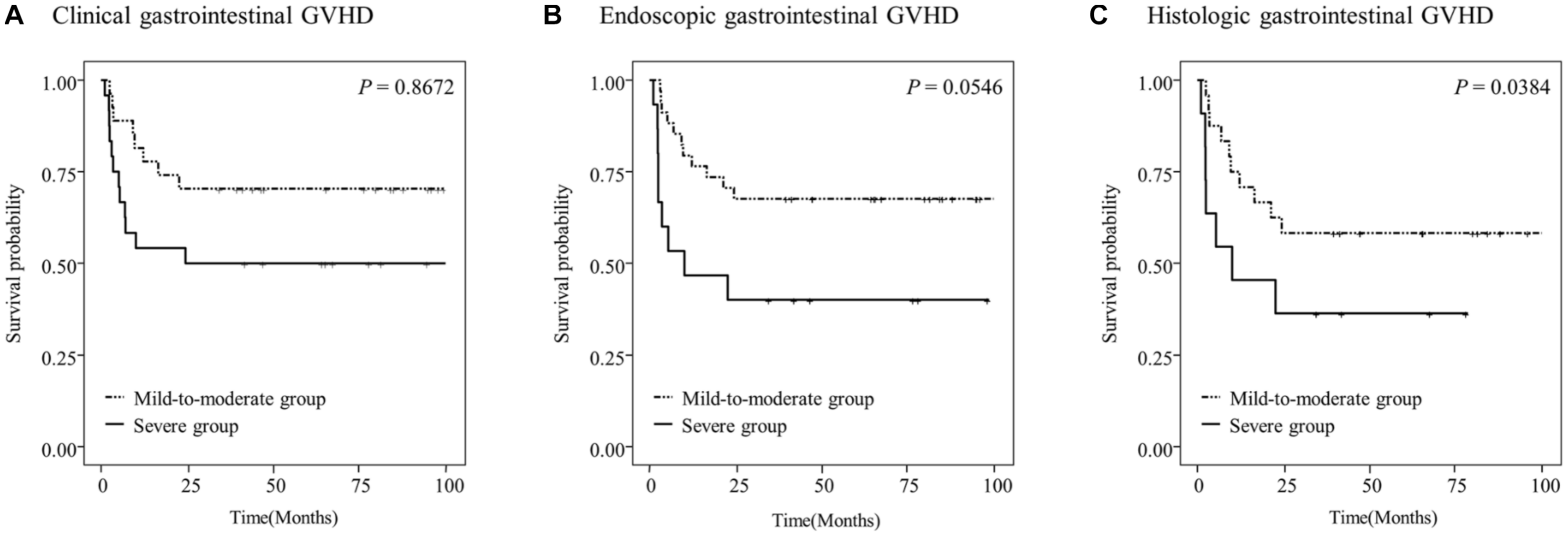
Overall survival (OS) of the study patients according to the clinical **(A)**, endoscopic **(B)**, or histologic **(C)** severity of gastrointestinal graft-versus-host disease (GVHD).

Kaplan–Meier survival curves were constructed to analyze the OS rates according to the assessment modality and severity of gastrointestinal GVHD (Figure 3). We compared the overall survival rate and cumulative incidence of NRM between the mild-to-moderate and severe groups, excluding the “absent” group, in patients with clinical (*n* = 51), endoscopic (*n* = 49), and histologic (*n* = 35) GVHD.

The 5-year OS rates in the study patients were 58.3 and 36.4% in the mild-to-moderate and severe histologic gastrointestinal GVHD groups, respectively (*p* = 0.0384; Figure 3C). However, no significant differences in the OS rates were observed according to the clinical and endoscopic severity of gastrointestinal GVHD (*p* > 0.05; Figures 3A,B).

**Figure 4:**
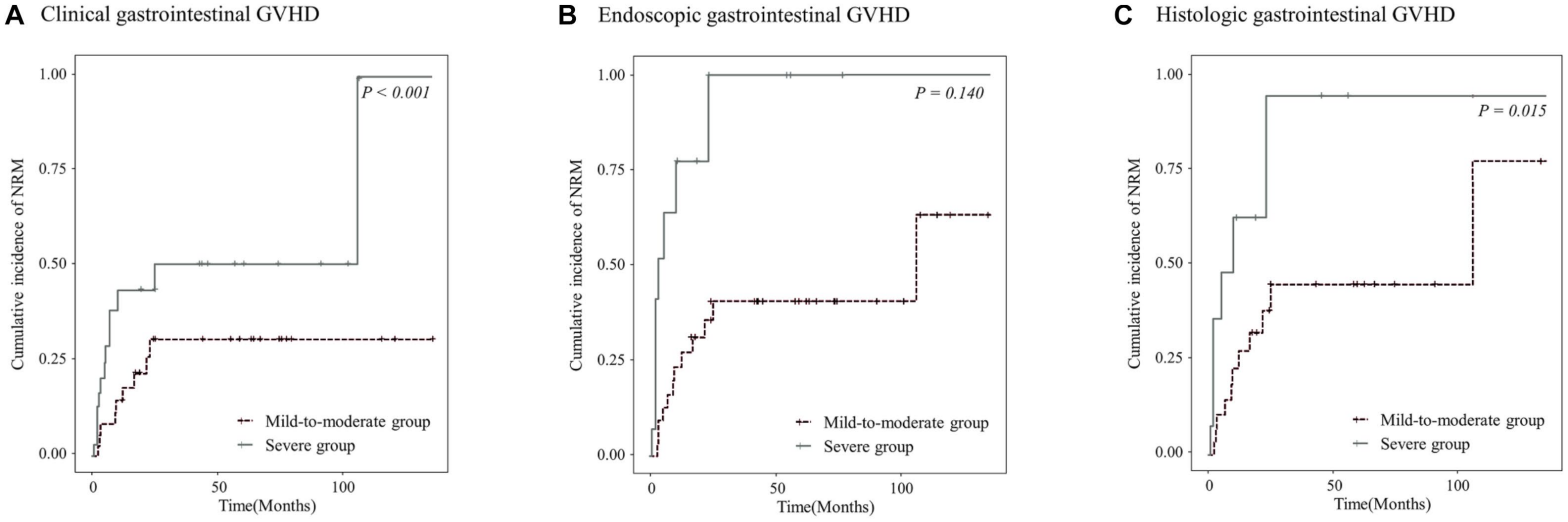
Cumulative incidence of non-relapse mortality (NRM) in the study patients according to the clinical **(A)**, endoscopic **(B)**, or histologic **(C)** severity of graft-versus-host disease (GVHD).

Patients with higher clinical and histologic grades of gastrointestinal GVHD had a higher cumulative incidence of NRM [*p* < 0.001 (Figure 4A) and *p* = 0.015 (Figure 4C), respectively]. However, no significant difference in the cumulative incidence of NRM was observed according to the endoscopic severity of gastrointestinal GVHD (*p* > 0.05; Figure 4B).

**Figure 5:**
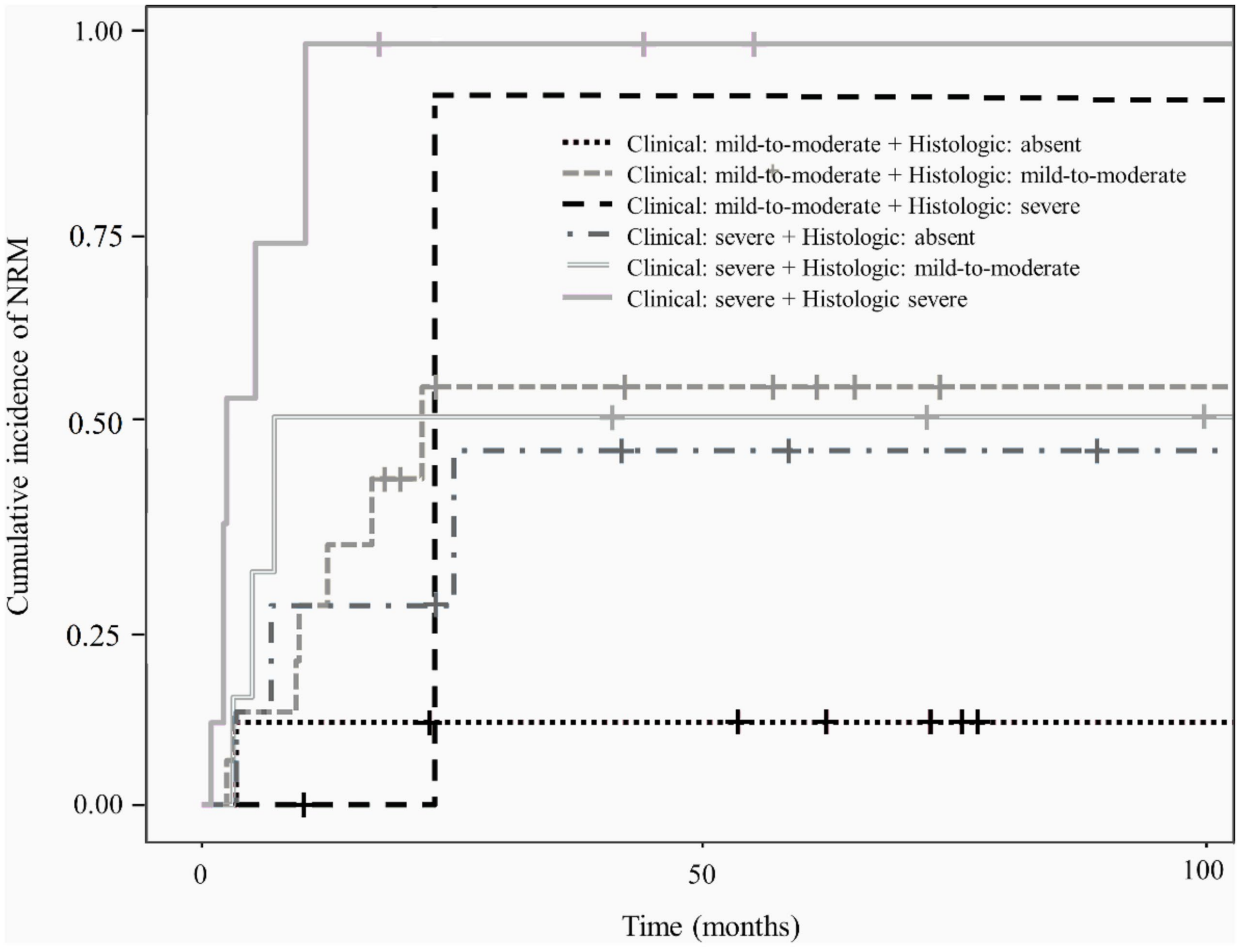
Cumulative incidence of non-relapse mortality according to the combined clinical and histologic severity of gastrointestinal graft-versus-host disease.

Figure 5 shows the cumulative incidence of NRM in patients with lower gastrointestinal GVHD stratified according to the combined clinical and histologic severity of GVHD. Even among patients with the same clinical severity of gastrointestinal GVHD, those with higher histologic grades had a higher cumulative incidence of NRM than those with lower histologic grades.

## Discussion

Although gastrointestinal GVHD is diagnosed on the basis of clinical manifestations, endoscopic and histologic examinations are required to exclude other conditions with similar symptoms (19, 20). Except for the purpose of diagnosis, the use of endoscopic and histologic grades of gastrointestinal GVHD is still controversial. In the current study, we demonstrated that the histologic severity of gastrointestinal GVHD was associated with both OS and NRM in pediatric patients treated with HCT. We also found that patients with mild-to-moderate histologic gastrointestinal GVHD, in addition to those with severe histologic gastrointestinal GVHD, had worse outcomes than patients without histologic gastrointestinal GVHD.

Endoscopic examinations can be difficult to perform in patients with a poor systemic status after HCT, especially in pediatric patients. Although the patient outcomes according to the clinical grade of gastrointestinal GVHD are well known, studies on prognostic differences according to endoscopic and histologic grades have been scarce, even in adult patients (21–26). In addition, although some studies have reported patient outcomes according to histologic grades, they mainly focused on grades 3–4 (4) and studies including all histologic grades are relatively rare. To our knowledge, this is the first study to address the significance of the histologic grades of gastrointestinal GVHD in predicting outcomes in pediatric patients treated with HCT.

The lower gastrointestinal tract is the organ most closely associated with mortality in patients with gastrointestinal GVHD (4, 27, 28). Although no standard method for the diagnosis of gastrointestinal GVHD has been established, the MAGIC guidelines identify histologic validation as the most definitive diagnostic test for patients with suspected gastrointestinal GVHD (5). Because the histologic findings of acute gastrointestinal GVHD can overlap with those of other disease entities, including infection and chemoradiation toxicity, the diagnosis is based on subtle histopathologic criteria.

Many investigators have attempted to develop histologic scoring systems for GVHD. Lerner et al., Melson et al., and Sale et al. proposed histologic criteria that have been widely accepted and used by clinicians and pathologists (18, 23, 29). The method that is most consistent with clinical data regarding diagnostic accuracy, treatment response, and outcomes is known as the Lerner method, which assigns four grades according to the degree of epithelial cell damage (30). In this scoring system for histologic severity, cases in which only the apoptotic bodies are visible are classified as grade 1, and cases with other additional histologic findings related to epithelial damage are systematically classified as grades 2–4. Because mild epithelial apoptosis can also be seen in other conditions such as inflammation, infection, and drug reactions (e.g., mycophenolate mofetil), the absent and mild-to-moderate categories of histologic gastrointestinal GVHD (grades 0–2) have received less attention than the severe category, and relatively few attempts have been made to analyze their association with patient outcomes.

False-positive results due to conditioning regimens have been reported to typically occur within 20 days of myeloablation (31). In this study, the median duration of endoscopy after HCT was 52 days, with no significant difference between groups based on histologic severity (*p* = 0.082). Furthermore, the proportions of patients receiving the conditioning regimens were not statistically different when the groups were stratified by histologic severity (*p* = 0.180). Therefore, the probability of a false-positive for gastrointestinal GVHD due to conditioning therapy in this study is considered low.

The histologic grades of gastrointestinal GVHD correlate with the clinical grades (23, 24, 32). Melson et al. reported that gastrointestinal crypt loss was highly correlated with the clinical grade of gastrointestinal GVHD based on the amount of diarrhea (23). In addition, Narkhede et al. found that the Spearman’s correlation coefficient between the histologic and clinical grades of gastrointestinal GVHD was 0.6, indicating a moderate association between the two components (24). Our study also revealed a strong correlation (ρ = 0.7, *p* = 0.0052) between the clinical and histologic grades of lower gastrointestinal GVHD, which is consistent with the results of previous studies. In addition, when the histologic grades were divided into severity categories, early onset of gastrointestinal GVHD symptoms, hematochezia, and severe clinical gastrointestinal GVHD were significantly more prevalent in patients with severe histologic gastrointestinal GVHD than in those with absent or mild-to-moderate histologic gastrointestinal GVHD. However, there was no additional association between the histologic severity of gastrointestinal GVHD and the prevalence of GVHD in other organs or overall GVHD grade.

The correlation between the histologic and endoscopic grades is controversial. Cruz-Correa et al. found a positive association between the endoscopic and histologic grades of gastrointestinal GVHD (odds ratio 12.0, 95% CI 3.9–37.2) (16). Meanwhile, Cheung et al. reported that endoscopic evaluation had low sensitivity and specificity for the diagnosis of gastrointestinal GVHD (33). In our study, the correlation between clinical and endoscopic grades was low (ρ = 0.3, *p* = 0.048) and that between endoscopic and histologic grades was moderate (ρ = 0.5, *p* = 0.001).

Several studies have shown that early onset of acute gastrointestinal GVHD, severe clinical gastrointestinal GVHD, hematochezia, mismatched donor type, and low albumin and high serum bilirubin levels are risk factors for mortality in patients with gastrointestinal GVHD (4, 34–36). Furthermore, in a study of 23 adult patients with gastrointestinal GVHD, a severe histologic grade was highly correlated with clinical grades 2–4, and patients with severe histologic gastrointestinal GVHD had a statistically significantly higher mortality rate than those with absent or mild-to-moderate histologic gastrointestinal GVHD (23). In addition, a study of 231 adult patients with GVHD confirmed that histologic grade was an important factor in mortality; however, this study included both skin and gastrointestinal GVHD cases (24).

In this study, severe clinical and histologic grades were associated with mortality, which is also consistent with the findings in adult studies (Table 3). Therefore, histologic findings are not only important clues for the diagnosis of gastrointestinal GVHD but also factors related to outcomes. As shown in Figure 2, histologic grade 3—4 is strongly associated with clinical grade 3–4, but lower histologic grades are distributed without significant association with clinical grade severity. Histologic grade was lower than the clinical grade in 68.6% of the patients, and patients with absent or mild-to-moderate histologic GVHD accounted for 78.5% of the study population. Therefore, low histologic grade does not equate to “mild” GVHD and it can be present even in patients with advanced clinical grade. Since the clinical and histologic severity of GVHD were found to influence the outcomes in the current study, we evaluated the cumulative incidence of NRM by combining these two factors (Figure 5). We observed that even among patients with the same clinical severity of gastrointestinal GVHD, the outcome was worse in those with higher histologic grades. In addition, patients with mild-to-moderate histologic gastrointestinal GVHD also had worse outcomes than those without histologic GVHD. This finding highlights that not only severe histologic grade but also mild-to-moderate histologic grade is a risk factor for worse outcomes (grade 1–2 vs. 0: HR 1.67, 95% CI 1.07–3.21, *p* = 0.0315). Therefore, histologic evaluation should be performed in addition to endoscopic evaluation in patients suspected of having acute gastrointestinal GVHD, not only for diagnostic purpose but also for prognostic evaluation.

The current grading system of histologic gastrointestinal GVHD reflect only short-term ongoing damage, and it is not immunologically proven why mild-to-moderate histologic GVHD is associated with worse outcomes. Recently, attempts have been made to overcome this limitation and to elucidate the relationship between the lower grades of the Lerner’s grading system and prognosis. Myerson et al. categorized low-level Lerner grade I into four activity grade categories based on the average frequency of apoptotic bodies (apoptotic index), which associated with therapeutic intervention (37). In the future, histologic grading of acute gastrointestinal GVHD, including immunologic features, should be investigated not only for the diagnosis of GVHD, but also for guiding treatment decisions and predicting outcomes.

The present study had several limitations. First, this was a retrospective study with certain disadvantages compared with prospective studies. However, all patients were hospitalized or attended the outpatient clinic at regular intervals during study period, allowing for consistent clinical evaluation and monitoring of acute gastrointestinal GVHD. Second, selection bias may have occurred by excluding patients with inadequate endoscopic and histologic evaluation. Most patients who were not included in this study were patients with mild gastrointestinal GVHD who responded well to glucocorticoids, the first-line therapy for GVHD, and were not candidates for endoscopy based on risk–benefit considerations at the time of treatment. Well-designed prospective studies of gastrointestinal GVHD in pediatric patients are needed to address the limitations of this study.

In conclusion, higher clinical and histologic grades of gastrointestinal GVHD are risk factors for NRM in pediatric patients treated with HCT. Furthermore, the histologic severity of gastrointestinal GVHD is a relevant factor affecting OS and NRM in these patients, with patients with mild-to-moderate or severe histologic gastrointestinal GVHD having worse outcomes than patients without histologic GVHD. These findings support the importance of assessing the histologic grade in the diagnostic evaluation of patients with clinical gastrointestinal GVHD. In addition, our results emphasize the prognostic value of the histologic grade of gastrointestinal GVHD.

## Data availability statement

The raw data supporting the conclusions of this article will be made available by the authors, without undue reservation.

## Ethics statement

The studies involving human participants were reviewed and approved by Institutional Review Board of Samsung Medical Center (file no. 2021–12-129). Written informed consent from the participants’ legal guardian/next of kin was not required to participate in this study in accordance with the national legislation and the institutional requirements.

## Author contributions

ESK, MJK, and KHY contributed to the conception and design of the study, interpreted the data, drafted the original manuscript, and revised the manuscript critically for important intellectual content. ESK and YK supported the data acquisition. ESK analyzed the data. All authors approved the final version to be published.

## Funding

This study was supported by a National Research Foundation of Korea (NRF) grant funded by the Ministry of Science and ICT (MSIT) of Korea (no. 2022R1F1A1073691).

## Conflict of interest

The authors declare that the research was conducted in the absence of any commercial or financial relationships that could be construed as a potential conflict of interest.

## Publisher’s note

All claims expressed in this article are solely those of the authors and do not necessarily represent those of their affiliated organizations, or those of the publisher, the editors and the reviewers. Any product that may be evaluated in this article, or claim that may be made by its manufacturer, is not guaranteed or endorsed by the publisher.
